# The importance of time‐to‐adjuvant treatment on survival with pancreatic cancer: A systematic review and meta‐analysis

**DOI:** 10.1002/cnr2.1390

**Published:** 2021-07-10

**Authors:** Kavin Sugumar, Jonathan J. Hue, Solanus De La Serna, Luke D. Rothermel, Lee M. Ocuin, Jeffrey M. Hardacre, John B. Ammori, Jordan M. Winter

**Affiliations:** ^1^ Department of Surgery University Hospitals Seidman Cancer Center Cleveland Ohio USA; ^2^ Case Comprehensive Cancer Center Case Western Reserve University School of Medicine Cleveland Ohio USA; ^3^ Department of Surgery, Division of Hepatobiliary Pancreatic Surgery, Atrium Health Charlotte North Carolina USA

**Keywords:** adjuvant chemotherapy, disease‐free survival, overall survival, pancreatic adenocarcinoma, time‐to‐treatment

## Abstract

**Background:**

While adjuvant chemotherapy benefits patients with pancreatic ductal adenocarcinoma (PDAC), the importance of the time to initiation of adjuvant therapy remains unclear.

**Aim:**

This study seeks to better understand whether the timing of postoperative chemotherapy initiation affects long‐term outcomes in PDAC.

**Methods and Results:**

A systematic literature search was performed in Medline, Embase, and Cochrane Library in March 2020. Studies focused on the association between the timing of adjuvant therapy on long‐term outcomes in resected PDAC patients were included. The impact of early and delayed therapy as defined by the respective studies was evaluated using forest plot analysis. Overall survival (OS) and disease‐free survival (DFS) served as primary endpoints. Out of 3099 published articles, 10 retrospective studies met inclusion criteria. Combined, these studies included clinical data of 13 344 patients. The cut off used to define “early” and “delayed” treatment groups varied in the included studies ranging from 3 to 12 weeks. Due to this heterogeneity, a sub‐group analysis of three time cut offs was performed: 3 to 5 weeks, 6 to 8 weeks, and 9 to 12 weeks. There was a significant decrease in OS and DFS when adjuvant therapy was delayed by 3 to 5 weeks after surgery (OS, pooled hazard ratio [HR] = 1.86, 95% confidence interval [CI] = 1.25‐2.78; DFS, pooled HR = 1.62, 95% CI = 1.12‐2.34). However, due to small sample size and limited studies in this subgroup analysis, the results may be indeterminate. There was no significant decrease in OS with delayed initiation of adjuvant therapy by 6 to 8 weeks and 9 to 12 weeks. Similarly, delay in adjuvant therapy beyond 3‐5 weeks.

**Conclusions:**

There was no conclusive evidence suggesting improved survival in patients starting treatment at various time cut offs. Studies investigating the extreme ends of the time‐to‐treatment spectrum may prove more informative.

## 1 INTRODUCTION

Pancreatic ductal adenocarcinoma (PDAC) is the most lethal common cancer. The overall five‐year survival remains just 10%.^1^, ^2^ Most patients present with unresectable disease. However, approximately 20% of patients have localized and resectable disease.^3^ The best chance of long‐term survival for such patients is resection combined with multi‐agent chemotherapy.^4^ Historically, chemotherapy was not considered as decidedly beneficial following surgical resection until the late 1990s.^5^ Based on the results of the european study group for pancreatic cancer (ESPAC‐1) trial in 2004,^6^ adjuvant chemotherapy using single agent 5‐fluorouracil (5‐FU) became the standard of care for resectable PDAC. A series of trials showing equipoise between gemcitabine and 5‐FU but a better toxicity profile in the adjuvant setting for gemcitabine shifted preference to this drug.^7^, ^8^, ^9^ Recent trials have determined a survival benefit for multi‐agent chemotherapy regimens when compared with single agent therapy.^5^

Despite the benefit of adjuvant chemotherapy, nearly half of the patients fail to receive additional therapy following resection, often due to a complicated postoperative course.^10^ Indeed, the time from a patients' operation until starting adjuvant therapy can vary widely.^11^, ^12^, ^13^, ^14^, ^15^, ^16^, ^17^, ^18^, ^19^, ^20^, ^21^, ^22^ Some patients may never recover enough to receive any amount of adjuvant treatment. Alternatively, patients may not tolerate multi‐agent chemotherapy after resection and instead receive less effective single agent therapy. The optimal timing for initiating adjuvant chemotherapy has not been rigorously evaluated. Most versed in the literature refer to a single post‐hoc analysis of a randomized trial which showed that adjuvant chemotherapy can be safely started up to 12 weeks post‐surgery.^12^

While there appears to be a benefit of adjuvant chemotherapy based on randomized adjuvant trial data, the impact of the timing of treatment in the general population is a question that requires further evaluation. Herein, we performed a meta‐analysis of the available literature to characterize the time from surgery to initiation of adjuvant therapy and determine the effect of delay on overall survival (OS) and disease‐free survival (DFS).

## 2 METHODS

### 2.1 Protocol and registration

This systematic review and meta‐analysis was conducted following the recommendations of the Preferred Reporting Items for Systematic Reviews and Meta‐analyses statement (PRISMA).^23^ It was registered in the International Prospective Register of Systematic Reviews (PROSPERO, registered as CRD42020170486).

### 2.2 Search strategy

Medline, Embase, and Cochrane Library databases were searched for publications from 1 January 1975 to 1 February 2020. The search query included pertinent strings of words including “pancreatic cancer AND adjuvant chemotherapy AND survival AND (time OR delay OR initiating OR start OR early).” Studies published in non‐English languages were excluded.

For inclusion, studies had to meet the following criteria: (a) observational cohort studies or randomized clinical trials, (b) patients diagnosed with clinically resectable PDAC and underwent resection, and (c) time duration from surgery to initiation of adjuvant chemotherapy were evaluated with either OS or DFS endpoints. Studies where patients received neoadjuvant therapy prior to surgery were excluded. Two independent individuals (KS and SS) reviewed titles and abstracts from the above‐mentioned databases and selected relevant publications. Studies fulfilling the inclusion criteria and abstracts lacking clear description of study parameters were acquired for a complete‐text evaluation. Any disagreement on eligibility for inclusion was reconciled by thorough discussion.

### 2.3 Data extraction and synthesis

The following data points were extracted: sample size, patient demographics, time cut‐offs delineating early and delayed initiation of adjuvant therapy groups, chemotherapy regimen used, median follow‐up period, usage of univariate, or multivariate survival analyses, and comparisons of median OS or DFS endpoints. The Newcastle‐Ottawa Scale (NOS) was used to ascertain the quality of observational studies and determine risk of bias.^24^ NOS scores ranged from 0 to 9 and can be categorized into three groups: very high risk (0‐3), high risk,^4^, ^5^, ^6^ and low risk of bias.^7^, ^8^, ^9^

Hazard ratio (HR) was used as the measure of effect for comparisons of OS and DFS between delayed and early treatment groups. Delayed chemotherapy was defined as chemotherapy started beyond a certain cutoff time period as described in the respective studies. For each study, the adjusted HR and 95% confidence interval (CI) were annotated, and SE was calculated from available data. If multivariable analyses were not performed, univariate HR was recorded. For studies where HR was not recorded, Kaplan‐Meir curves were digitalized using Webplot digitalizer software^25^ and HR and 95% CI was estimated.^26^ For both OS and DFS, each publication was weighted as a function of the inverse variance of each effect size and forest plots were constructed. Cochrane Chi^2^ and I^2^ statistics were used to assess homogeneity for each outcome. Studies were considered to have significant heterogenicity when Chi^2^ *P*‐value was less than .1 and I^2^ was greater than 50%.

The pooled HR for OS and DFS with early vs delayed adjuvant therapy was calculated either using the fixed effects model/Manzel‐Haenzel method or random effects model/DerSimonian‐Laird method based on heterogeneity of the included studies. Random effects method was used when I^2^ was greater than 50%.

The publication bias was evaluated using Egger's linear regression and funnel plot analysis to illustrate asymmetry between studies. Studies were considered to have significant publication bias if *P*‐value was less than .05. The GRADE approach was utilized to evaluate the quality of evidence of this meta‐analysis. The assessment includes risk of bias, imprecision, inconsistency, indirectness, publication bias, magnitude of effects, dose‐response relations, and impact of residual confounding and bias.^27^ Using the above parameters, the GRADE certainty rating is graded as very low, low, moderate, and high. The GRADEpro Guideline Development Tool (McMaster University, 2020, developed by Evidence Prime, Inc.) was used to calculate and tabulate the GRADE certainty rating.

All statistical analyses were performed with Review Manager (RevMan) (computer program) Version 5.3 (Copenhagen: The Nordic Cochrane Centre, The Cochrane Collaboration, 2014) and StateSE Version 16 software (StataCorp. 2019. *Stata Statistical Software: Release 16*. College Station, TX: StataCorp LLC).

## 3 RESULTS

**FIGURE 1:**
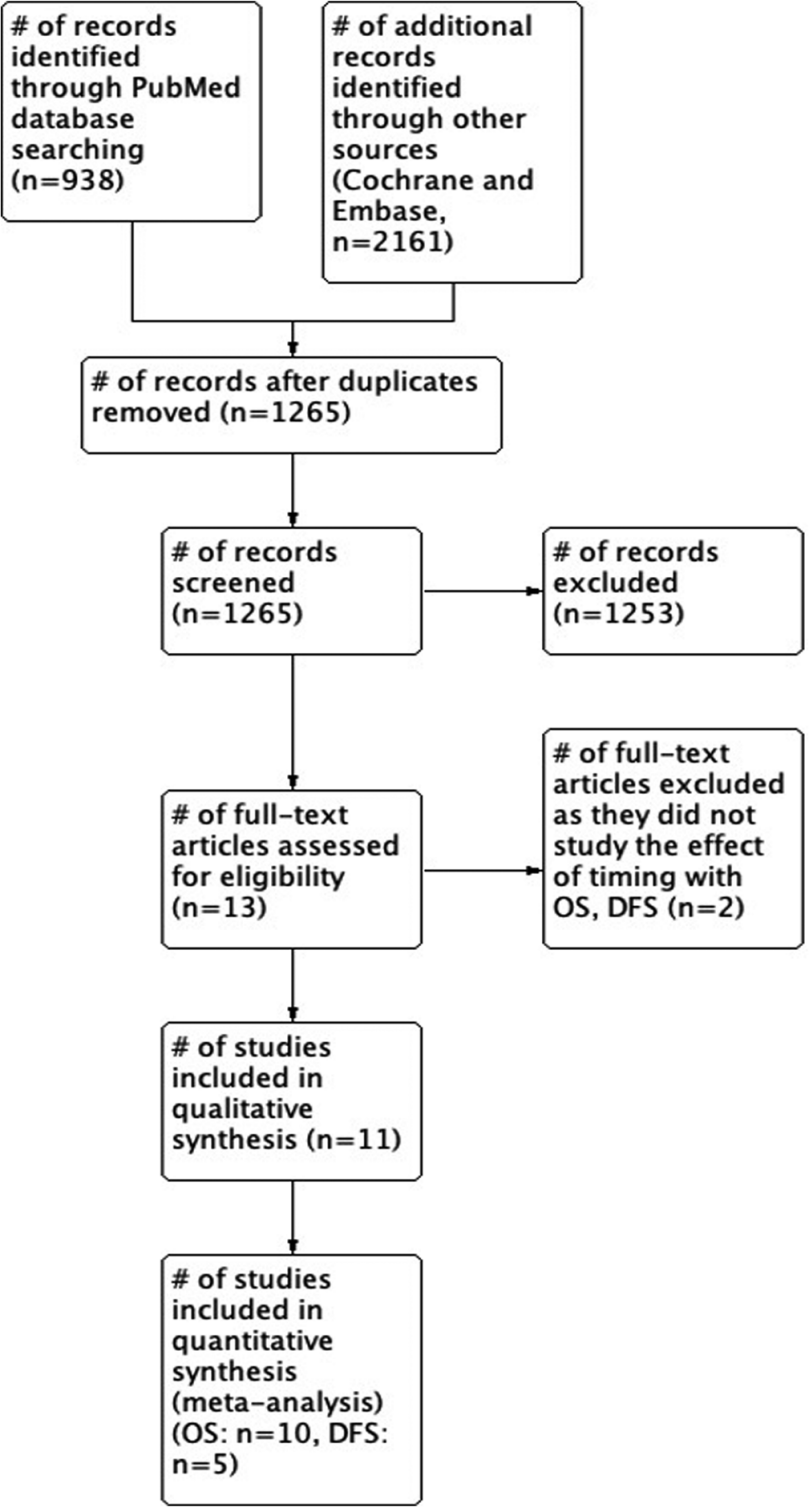
Study flow diagram

The literature search identified 3099 studies (Figure 1). After screening of titles and abstracts, complete text of 13 studies were obtained for further review. Of the 13 studies, two were excluded as they did not study the effect of timing of adjuvant therapy on long‐term outcomes. In total, we included 11 publications for descriptive analysis in this study. There were no non‐English studies on the subject topic. Of these 11 studies, one utilized three time periods and was excluded. Ten studies utilized two time periods: early and delayed‐treatment groups. These 10 studies were further used for performing the meta‐analysis.

### 3.1 Study characteristics

Table 1 shows the characteristics of the included studies. 91% of studies were retrospective cohort studies (Murakami 2013, Patel 2015, Saeed 2015, Yabusaki 2016, Mirkin 2016, Xia 2017, Kim 2017, Lee 2017, Ma 2019, White 2019) and one study was a post‐hoc analysis of a phase III randomized clinical trial (Valle 2014). Studies reported results using a variety of adjuvant chemotherapy regimens. The most common drugs used were gemcitabine monotherapy, 5‐fluorouracil (5‐FU) monotherapy, FOLFIRINOX (folinic acid, 5FU, oxaliplatin, irinotecan), capecitabine, and S‐1 (tegafur, gimeracil, oteracil). Three studies excluded patients who underwent radiation therapy in addition to adjuvant therapy (Murakami 2013, Valle 2014, Mirkin 2016). The time from surgery to initiation of chemotherapy used to delineate early vs delayed ranged from 20 days to 12 weeks across studies. One retrospective study (Ma 2019) utilized three‐time intervals of <4 weeks, 4 to 8 weeks, and >8 weeks, and was excluded.

**TABLE 1:** Study characteristics

Author	Murakami	Valle	Patel	Mirkin	Saeed	Yabusaki	Kim	Lee	Xia	Ma	White
Year	2013	2014	2015	2016	2016	2016	2017	2017	2017	2019	2019
Country	Japan	UK	USA	USA	USA	Japan	Korea	Korea	USA	USA	USA
Study type	Retrospective	Prospective (Phase III)	Retrospective	Retrospective	Retrospective	Retrospective	Retrospective	Retrospective	Retrospective	Retrospective	Retrospective
Institution	Single	Multi	Single	Single	Multi	Single	Single	Multi	Multi	Single	Single
Administrative database	No	No	No	NCDB	State registry	No	No	No	No	NCDB	NCDB
Sample size	103	985	30	4392	420	79	113	309	488	7548	10 221
Median age, years	69	63	58.5	65	63.5	64	63	61.3	67	67	66
Stage	I, II, III, IV	I, II, III, IV	I, II, III	I, II, III	I, II, III	I, II, III	NR	I, II, III	I, II, III	I, II	I, II, III
Median Follow up (months)	47.1	58	22	58	19.3	24.5	20.3	28	NR	38.6	20
Groups divided	< & > 20 d	< & > 8 wk	< & > 8 wk	< & > 12 wk	< & > 8 wk	< & > 8 wk	< & > 5 wk	< & > 6 wk	< & > 6 wk	< 4, 4‐8.4, and >8.4 wk	< & > 66 d
CT administered	Gem and S‐1	Gem, 5‐FU	Gem, Cap, 5‐FU	NR	NR	Gem and S‐1	Gem, 5‐FU	Gem, 5‐FU	Gem	Gem, 5‐FU, FOLFIRINOX	NR
CRT	No	No	Yes	No	Yes	Yes	Yes	Yes	Yes	Yes	Yes
Multivariate analysis	Yes	Yes	No	Yes	Yes	Yes	Yes	Yes	Yes	Yes	Yes
Median survival (Early vs late, months)	NR	22.6 vs 24.2	18 (overall)	22 vs 20.8	20 vs 19	RDI > 80% 45 vs 43, RDI < 80% 25 vs 29	39 vs 21	33 vs 38	24.3 vs 28.5	20.6, vs 22 vs 20.4	21.8
Disease‐free survival (Early vs late, months)	NR	13 vs 14	17 (overall)	NR	NR	NR	18 vs 10	NR	13.6 vs 16	NR	NR

Abbreviations: Cap, Capecitabine; Gem, gemcitabine; NR, not recorded; RDI, radiation dose intensity.

### 3.2 Timing of adjuvant chemotherapy on OS

Ten studies evaluated the effect of a delay in initiating adjuvant chemotherapy on OS. In total, these studies included 13 344 patients. The number of patients was nearly equally distributed between early and delayed‐treatment groups.

**FIGURE 2:**
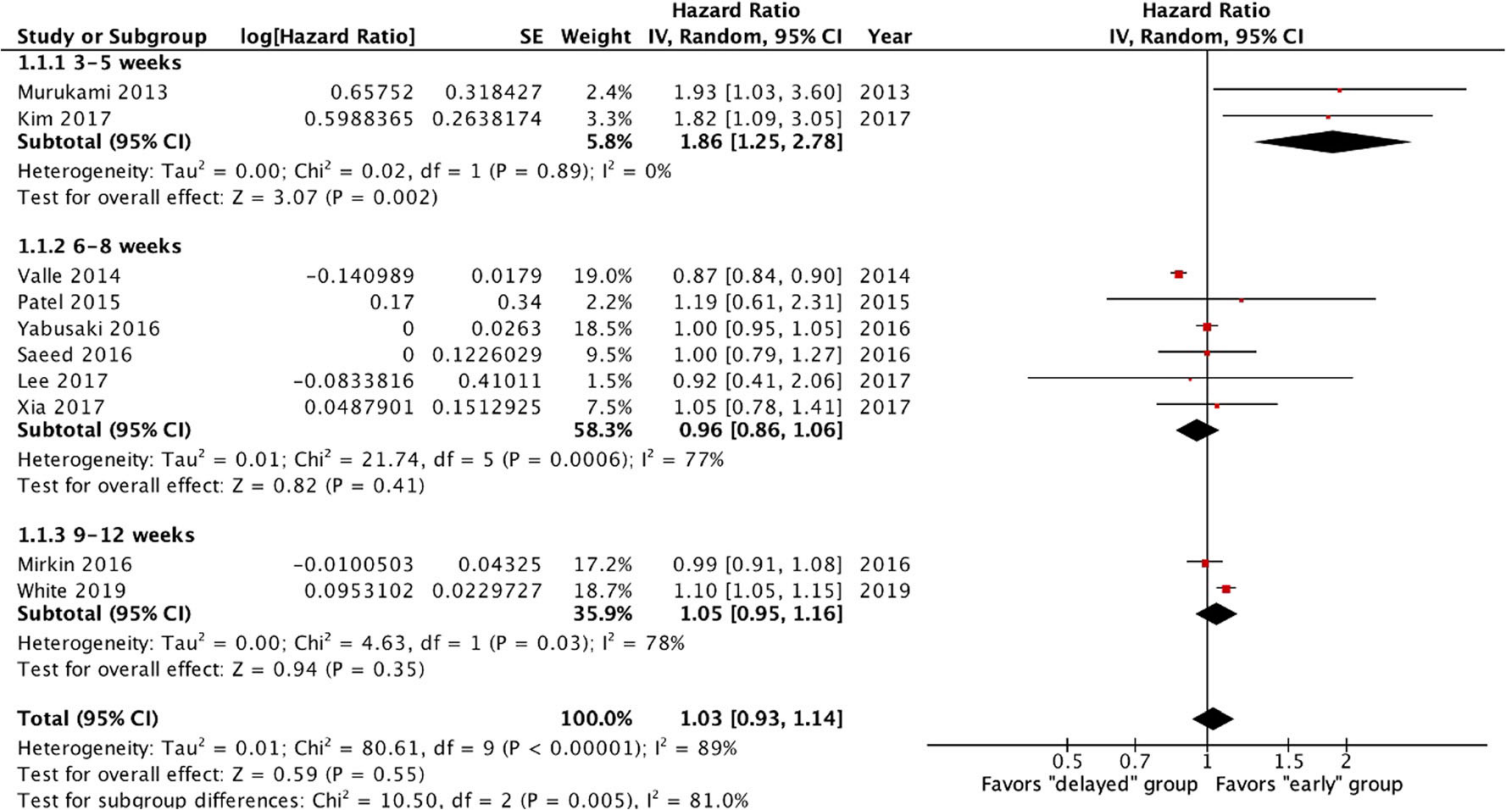
Forest plot of delayed vs early initiation of adjuvant chemotherapy on overall survival (OS)

**FIGURE 3:**
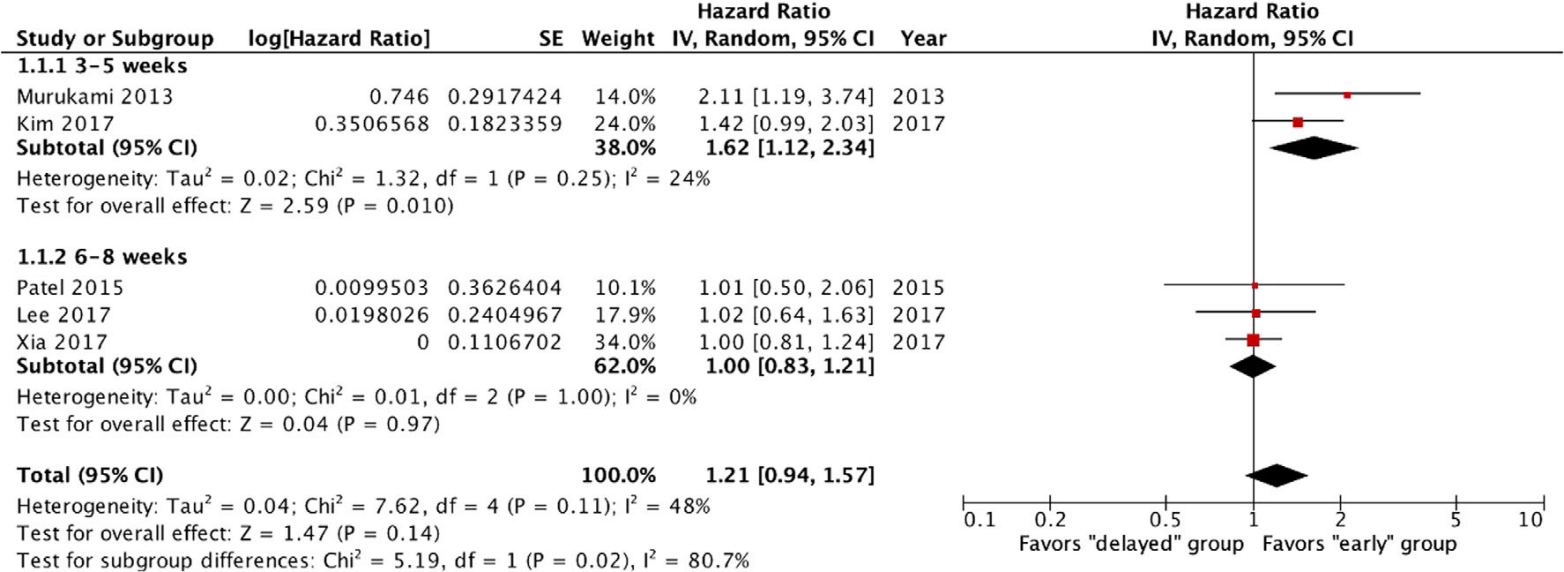
Forest plot of delayed vs early initiation of adjuvant chemotherapy on disease‐free survival (DFS)

Due to the varying time cut‐offs used in the 10 studies to define early and late treatment groups, subgroup analyses were performed for the following cut‐offs: 3 to 5 weeks, 6 to 8 weeks, and 9 to 12 weeks. Given the heterogeneity in the definition of the study groups, random effects model was chosen for all further analyses. There was a significant decrease in OS in the two studies where adjuvant therapy was delayed by 3 to 5 weeks after surgery (pooled HR = 1.86, 95% CI = 1.25‐2.78) (Figure 2). However, there was no significant decrease in OS with delayed initiation of adjuvant therapy by 6 to 8 weeks (pooled HR = 0.96, 95% CI = 0.86‐1.06) or 9 to 12 weeks (pooled HR = 1.05, 95% CI = 0.95‐1.16, Figure 3).

### 3.3 Timing of adjuvant chemotherapy on DFS

Five studies examined the effect of delay in adjuvant therapy on DFS and included 1043 patients. Similarly, due to the varying time cut‐offs used to define early and late treatment groups, subgroup analyses were performed for the following cut‐offs: 3 to 5 weeks and 6 to 8 weeks. There was a significant decrease in DFS when adjuvant therapy was delayed by 3 to 5 weeks after surgery (pooled HR = 1.62, 95% CI = 1.12‐2.34) (Figure 2). Again, there was no significant decrease in DFS with delayed initiation of adjuvant therapy by 6 to 8 weeks (pooled HR = 1, 95% CI = 0.83‐1.21).

### 3.4 Risk of bias (Newcastle‐Ottowa score)

**FIGURE 4:**
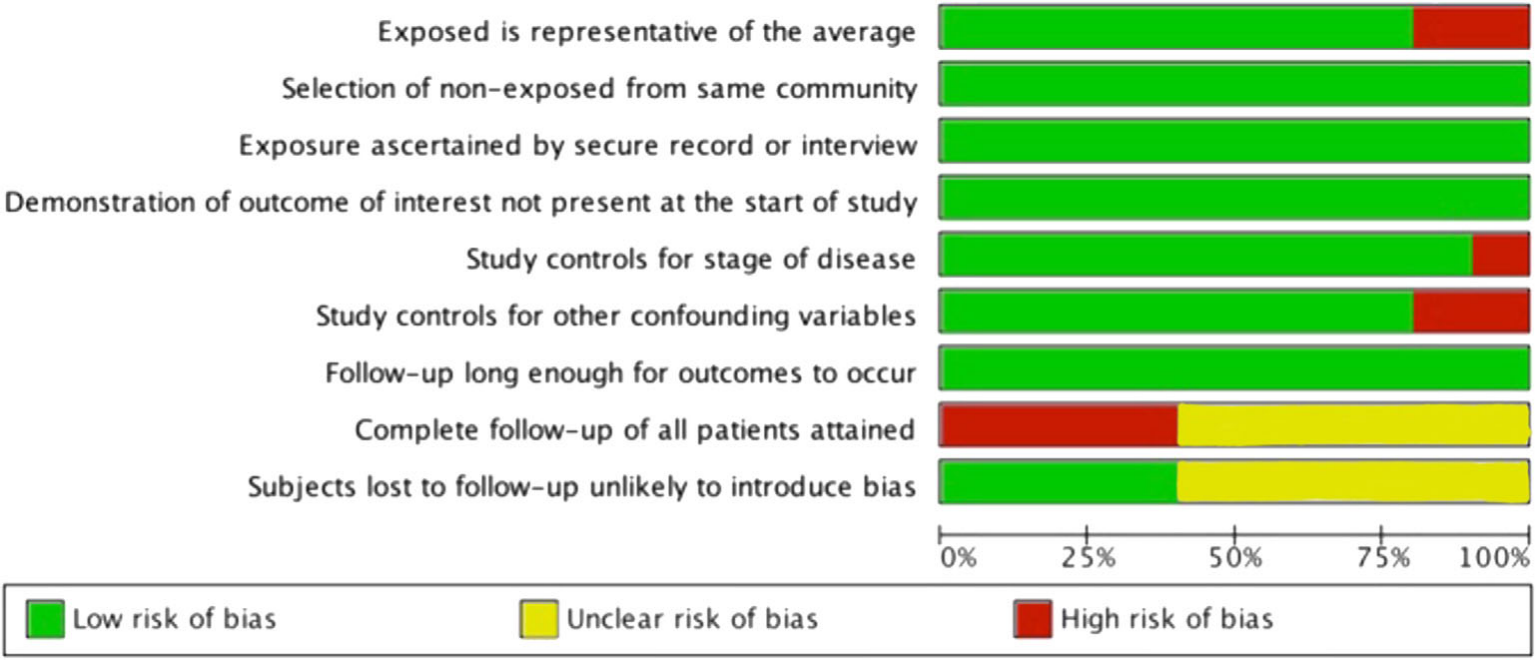
Risk of bias graph: review authors' judgments about each risk of bias item presented as percentages across all included studies

Six studies had a NOS score of 7 or higher (high quality observational studies with low risk of bias) including Valle 2014, Saeed 2016, Yabusaki 2017, Lee 2017, Xia 2017, and White 2019. The median NOS score was 7 (range: 6‐8) (Table 2, Figure 4). This indicates a high study quality among the included studies. All studies had equally distributed “early” and “delayed” treatment cohorts from the same patient population. Most of the studies adjusted for various confounding variables including stage, age, and other pathological findings. Only four studies reported the number of patients lost to follow‐up. However, this number was very small to result in any potential bias. The remaining six studies did not report the patients lost to follow‐up, which could contribute to selection bias.

**TABLE 2:** Newcastle‐Ottowa score for ascertaining risk of bias among included studies

	Item	Murakami et al^11^	Valle et al^12^	Patel et al^22^	Mirkin et al^13^	Saeed et al^14^	Yabusaki et al^15^	Kim et al^16^	Lee et al^17^	Xia et al^18^	White et al^21^
**A**	**Selection**										
1.	Exposed is representative of average	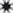	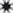		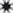	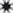		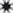	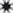	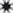	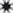
2.	Selection of comparison group from same community	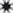	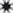	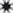	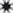	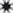	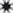	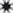	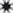	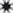	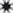
3.	Exposure ascertained by secure record or interview	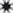	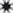	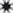	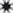	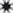	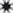	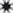	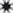	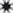	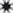
4.	Demonstration of outcome of interest not present at the start of study	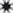	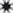	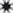	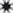	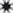	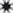	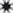	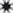	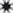	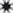
											
**B**	**Comparability**										
1.	Study controls for stage of disease	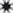	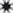	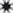	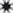	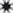	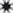		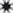	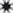	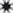
2.	Study controls for other confounding variables	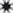	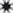			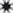	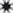	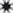	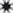	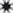	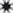
											
**C**	**Outcome**										
1.	Follow‐up long enough for outcomes to occur	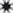	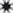	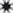	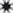	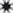	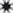	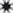	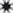	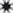	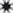
2.	Complete follow‐up of all patients attained	?			?			?	?	?	?
3.	Subjects lost to follow‐up unlikely to introduce bias	?	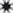	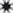	?	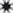	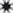	?	?	?	?
											
	Total Score	6	8	6	6	8	7	6	7	7	7

*Note*: 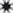 contributes one point to the final score. ? unclear.

### 3.5 Publication bias

**FIGURE 5:**
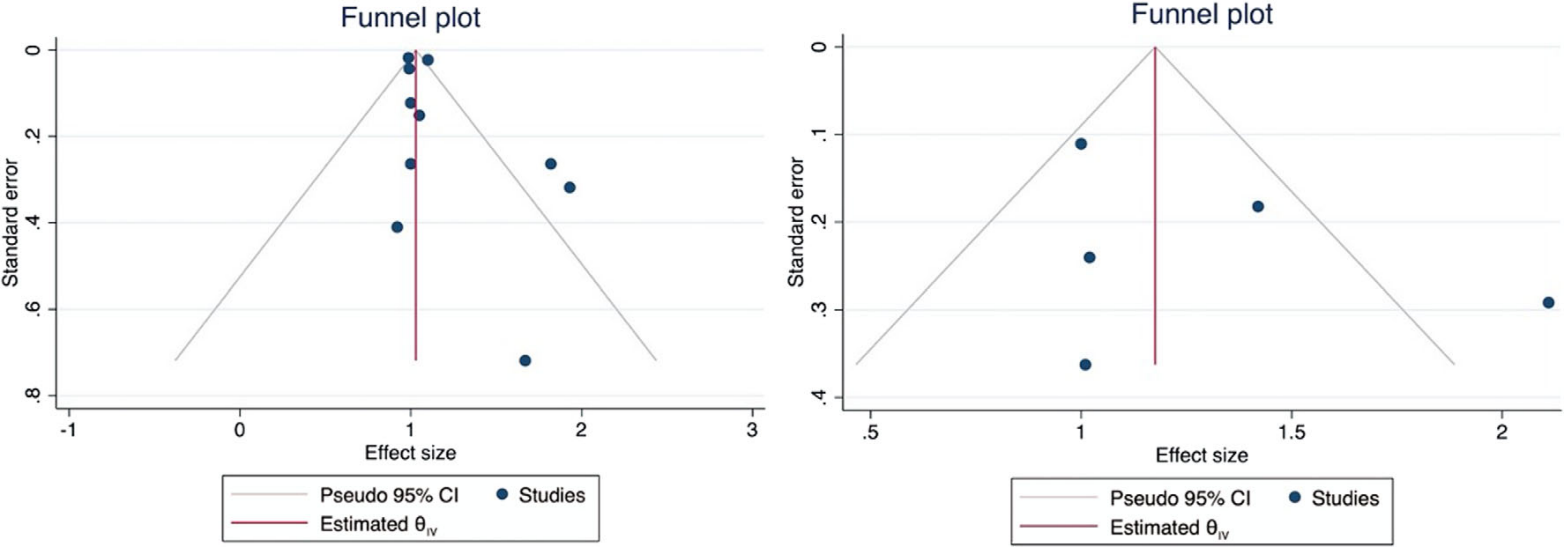
Funnel plot showing the relationship of hazard ratio (HR) and SE on A, overall survival (OS) and B, disease‐free survival (DFS)

The funnel plot was used to study the degree of asymmetry of individual study results around the pooled HR for OS (Figure 5A). Asymmetry was detected which was further analyzed using the Egger method. There was significant asymmetry in study results (*P* = .02) which indicates a publication bias. A funnel plot was also constructed for DFS and was found to be symmetrical (Figure 5B), as confirmed by Egger test (*P* = .59).

### 3.6 GRADE certainty rating

The GRADE certainty assessment is shown in Table 3. The GRADE rating indicated very low and low quality of evidence for the meta‐analyses evaluating OS and DFS, respectively.

**TABLE 3:** GRADE approach to ascertain certainty of evidence

GRADE certainty assessment						
Participants (studies) Follow up	Risk of bias	Inconsistency	Indirectness	Imprecision	Publication bias	Overall certainty of evidence
**Overall survival (follow up: median 2.75 y)**						
13344 (10 observational studies)	not serious	not serious	not serious	not serious	publication bias strongly suspected^a^	⊕◯◯◯VERY LOW
**Disease‐free survival (follow up: median 2.44 y)**						
1043 (5 observational studies)	not serious	not serious	not serious	not serious	none	⊕⊕◯◯LOW

Abbreviations: CI, confidence interval; HR, hazard ratio.

^a^ Publication bias was assessed using Funnel plots and the Egger test.

## 4 DISCUSSION

The role of adjuvant chemotherapy for PDAC has been extensively studied over the past two decades. As the majority of PDAC are usually associated with micrometastases at presentation, even at the clinically resectable and localized stage,^28^ the main goal of adjuvant chemotherapy is to treat these micrometastases in order to delay relapse. Collectively, randomized clinical trials show that multi‐agent chemotherapy offers the best chance of long‐term survival after pancreatic resection,^4^ and this is reflected in the National Comprehensive Cancer Network (NCCN) guidelines. However, the question of chemotherapy timing remains unclear. Many of these landmark trials studying postoperative chemotherapy in PDAC either did not comment on the timing of therapy initiation or utilized wide time intervals in which the chemotherapy could be started. As a benchmark, randomized phase III trials almost universally initiate therapy within 12 weeks of resection. This study reviews the current literature and provides a meta‐analysis to investigate the impact of the timing of adjuvant chemotherapy initiation on survival for PDAC.

Since chemotherapy offers a survival benefit (as compared to no chemotherapy), it is logical to suppose a benefit to early initiation of chemotherapy recovery from surgery. A delay in time to adjuvant chemotherapy has been associated with worse survival in certain malignancies like head and neck, colorectal, cervical, and breast cancers.^29^, ^30^, ^31^, ^32^, ^33^ However, studies looking at the same in pancreatic cancer have yielded mixed results.

From the current meta‐analysis, Murakami et al^11^ concluded that when adjuvant therapy was initiated within 20 days of surgery, it was associated with better OS. Using data from the ESPAC‐3 trial, Valle et al compared patients who received single‐agent gemcitabine or 5‐FU after resection. Initiation of chemotherapy before or after 8 weeks postoperatively was not shown to have an effect on OS. Oddly, subgroup analysis of patients who did not complete adjuvant therapy (less than six cycles) had a survival advantage if chemotherapy was delayed.^11^ The authors attributed this to the insufficient duration of recovery from immune system suppression following surgery.^34^, ^35^, ^36^ In 2015, Patel et al^22^ published a report showing that there was no association between progression‐free survival or OS and the time to start adjuvant therapy (chemotherapy or chemoradiation). Similarly, Mirkin et al^13^ concluded that early initiation of chemotherapy did not impact survival and recommended adjuvant chemotherapy be delayed until patients were healthy enough to tolerate the therapy.

Two additional reports from 2016 found no differences in OS when comparing those who started chemotherapy within 8 weeks of surgery and those who started treatment after the eight‐week timepoint.^14^, ^15^ Lee et al^17^ reported that early vs late initiation of adjuvant therapy, defined as before or after 6 weeks postoperatively, did not impact OS. However, the authors also commented that patients who were able to complete therapy had a significant survival advantage. A multi‐institutional study from 2017 showed that timing of adjuvant therapy (before or after 12 weeks postoperatively) did not affect survival, while those who received surgery alone had a reduced OS compared with patients who received adjuvant chemotherapy at any time.^18^

Finally, a recent meta‐analysis showed no differences in survival when comparing patients who started chemotherapy within 6 to 8 weeks to those who started after 8 weeks.^20^ The aforementioned meta‐analysis differs from the present one in that it was more limited in scope. Studies evaluating TTT with cut offs other than 6 to 8 weeks were not included. The study only included six studies in their analysis (vs 10 here). Also, DFS was not considered as an outcome. A thorough risk of bias analysis and effect on the certainty of evidence were not investigated.

Contrary to the previously mentioned papers, a few studies did find an association between timing of chemotherapy and survival. For instance, Kim et al^16^ reported that patients who received treatment within 5 weeks of surgery had significantly better OS, as compared to those who started therapy after this cutoff. However, patients who were not able to complete their adjuvant treatment regimen faired significantly worse. A large study using the NCDB showed that early adjuvant chemotherapy (started within 4 weeks of surgery) and delayed initiation (started after 8.4 weeks) had higher mortality rates compared to those who started chemotherapy between 4 and 8.4 weeks.^19^ This study on its own might suggest that initiation too early may put the patient at risk, and that a “sweet‐spot” timeframe was possible. White et al^21^ performed a propensity score matched analysis of NCDB patients and concluded that patients who received chemotherapy before 66 days had a better survival advantage.

From our meta‐analysis, patients who received adjuvant therapy 3 to 5 weeks after surgery had decreased DFS and OS. However, this sub‐group analysis suffers from the limitation of small sample size derived from just two retrospective cohort studies. Also, our results demonstrate a wide confidence interval, which could suggest a relatively imprecise estimate of outcome. We found no significant difference in DFS and OS between “early” and “delayed” adjuvant therapy groups at 6 to 8 week and 9 to 12‐week cut‐offs.

Despite the fact that all of the included studies were observational, NOS grading suggested that most of the studies were of high quality. The GRADE approach showed very low and low certainty of evidence in the studies evaluating OS and DFS, respectively. This low level of evidence is primarily due to the retrospective observational study design of nearly all studies. The presence of publication bias associated with studies evaluating OS further reduces the accuracy of the data.

There could be many possible reasons for the findings from this study. As evident, the available literature on timing of adjuvant chemotherapy after pancreatic resection utilizes various timepoints to define early vs late initiation. Also, the studies had diverse populations of pathologically diagnosed stages I, II, III, and IV PDAC patients. Multiple chemotherapy regimens were utilized between studies, and even within studies. All of these factors could contribute to the heterogeneity of results. There seems to be significant asymmetry of study results on Funnel plot analysis, which was confirmed by the Egger test. This may suggest a publication bias in studies focused on the subject.

Moreover, these data are neither prospective, nor randomized. Therefore, they are prone to key selection biases. For instance, patients who have not progressed after a delay in therapy likely have favorable tumor biology. Had they progressed with recurrence and metastatic, they would not have been included in the delayed cohort, since the treatment would be considered palliative and not adjuvant. This would positively impact survival outcomes in the delayed group. In contrast, more patients in the early group likely have unfavorable biology for the same reasons. Many of these patients would progress through chemotherapy and relapse quickly. On the other hand, many patients who experience a delay in treatment likely had the most trouble recovering from surgery and would be expected to negatively impact patients in the delayed treatment group.

It is also important to recognize that modern chemotherapy, albeit beneficial, only offers a marginal survival advantage. OS improvements are typically between 2 and 4 months in most randomized trials, while DFS is around 7 months.^37^ The recent PRODIGE trial testing FOLFIRNOX is a noteworthy exception with a much stronger reported benefit.^38^ When the signal is so small between treatment and no‐treatment groups and requires several hundred patients to even detect a difference, we should not be surprised that a delay of a few months does not translate into a detectable difference. Finally, category distinctions with a single, dichotomous cutoff point (eg, > or <8 weeks) may not prove prognostic because they do a poor job of distinguishing treatment effects across subgroups. If most of the patients begin therapy around 8 weeks, one would not expect to detect a signal between patients who start treatment at 9 weeks vs 7 weeks. Rather, studies looking at extreme ends of the treatment spectrum may prove more informative.

The strongest proponents of early initiation of chemotherapy often favor the use of neoadjuvant therapy for localized PDAC.^39^ They argue that this approach guarantees rapid time to systemic treatment. Currently, there is not enough evidence to support this argument. The best available literature to date studying multi‐agent chemotherapy for resectable PDAC occurs in the adjuvant setting.^38^, ^40^, ^41^ Moreover, early systemic treatment in the SWOG 1505 neoadjuvant trial did not translate into superior survival outcomes (as compared to historical data from adjuvant trials) that support the theoretical arguments.^42^ Taken together, the present study in combination with other informative and relevant studies in the literature have not proven that time to treatment (chemotherapy or surgery) is critical. However, the relatively rapid progression between stages that is observed with PDAC strongly suggests that time‐to‐treatment is of the essence, and that the inability to detect a signal is related to the above‐mentioned limitations and selection biases. After all, localized PDAC treated with surgery alone has a median survival of roughly 15 months, giving some sense that untreated localized PDAC progresses to metastatic disease in a year timeframe.^43^

The study has technical limitations that also deserve mention. The included studies had varying definitions for “early” and “delayed” treatment groups. Though we performed subgroup analyses and meta‐regression to account for the significant heterogeneity of studies, certain subgroups consisted of statistically less complex or smaller studies, which may not provide accurate estimates of the actual effects. Certain studies reported adjusted HR whereas others had unadjusted HR, which could lead to bias on the pooled analysis. In the studies that did not provide HR, there may be a possibility of error in synthesizing the HR from the Kaplan Meier curves. Also, this systematic review and meta‐analysis is prone to potential errors in search methodology, selection, and reporting bias. Given the paucity of prospective research in this subject topic, the certainty of evidence of this meta‐analysis is low. An ideal study would involve a prospective, multi‐center, randomized clinical trial. However, feasibility of such a prospective study would be questionable, provided that ethical concerns in regard to delaying adjuvant therapy in this subset of patients.

## 5 CONCLUSION

Our meta‐analysis shows that there was no conclusive evidence suggesting improved survival in patients starting treatment at various time cut offs. Given the paucity of prospective studies, the results need to be cautiously interpreted. Further multi‐institutional studies utilizing similar chemotherapy regimens that compare the extreme ends of the treatment spectrum are required. Based on our understanding of the natural history and biology of PDAC, time‐to‐treatment should be optimized with a goal to deliver treatment as soon as the patient is clinically recovered from surgery and considered to be fit enough to tolerate chemotherapy.

## CONFLICT OF INTEREST

The authors declare no conflicts of interest.

## AUTHOR CONTRIBUTIONS

*Conceptualization; data curation; formal analysis; investigation; methodology; project administration; resources; software; writing‐original draft*, K.S.; *Methodology; project administration; resources; software; supervision; validation; visualization; writing‐review & editing*, J.H.; *Data curation; formal analysis; investigation; methodology; project administration; resources; software; writing‐review & editing*, S.D.L.S.; *Investigation; methodology; project administration; supervision; validation; visualization; writing‐review & editing*, L.R.; *Investigation; methodology; project administration; software; supervision; validation; visualization; writing‐review & editing*, L.O.; *Investigation; methodology; project administration; software; supervision; validation; visualization; writing‐original draft; writing‐review & editing*, J.H. and J.A.; *Conceptualization; methodology; project administration; resources; supervision; validation; writing‐original draft; writing‐review & editing*, J.W.

## ETHICAL STATEMENT

This study was approved and registered as a systematic review in the PROSPERO database on 04/28/2020 (Registration number: CRD42020170486).

## Data Availability

The data that support the findings of this study are available from the corresponding author upon reasonable request.
